# Moss Bags as Biomonitors of Atmospheric Microplastic Deposition in Urban Environments

**DOI:** 10.3390/biology12020149

**Published:** 2023-01-18

**Authors:** Carter Bertrim, Julian Aherne

**Affiliations:** School of Environment, Trent University, Peterborough, ON K9L 0G2, Canada

**Keywords:** microfibres, active biomonitoring, *Pleurozium schreberi*, Ontario, Canada

## Abstract

**Simple Summary:**

The atmosphere is an important transport pathway of microplastics to remote and urban environments. We assessed the efficacy of moss bags as an active biomonitoring technique for atmospheric microplastic deposition. Microplastics were observed in all moss bags deployed along an urban intensity gradient; moss bags exposed in the most densely populated and trafficked areas accumulated a higher number of microplastics compared with those exposed in low-density areas.

**Abstract:**

Microplastics (plastic particles <5 mm) were first identified in the environment during the 1970s and have since become ubiquitous across every environmental compartment. However, few studies have focused on atmospheric microplastics, and even fewer have used biological monitoring to assess their atmospheric deposition. Here, we assess the efficacy of moss bags as an active biomonitoring technique for atmospheric microplastic deposition. Moss (*Pleurozium schreberi*) bags were exposed in duplicate at nine deployment sites across a gradient of urban intensity in southern Ontario, Canada. A total of 186 microplastics (mp) were detected in the moss bags, resulting in a mean accumulation of 7.9 mp g^−1^ dry weight moss across all sites during the exposure period (45 days). The median microplastic length was 0.56 mm (range 0.03–4.51 mm), and the dominant microplastic type was fibres (47%), followed by fragments (39%). Microplastic accumulation significantly increased with urban intensity, ranging from 3.7 mp g^−1^ in low-density suburban areas to 10.7 mp g^−1^ in densely populated and trafficked urban areas. In contrast, microfibres by proportion dominated in suburban (62%) compared with urban areas (33%). Microplastic deposition was estimated to range from 21 to 60 mp m^−2^ day^−1^ across the nine deployment sites. The results suggest that moss bags may be a suitable technique for the active biomonitoring of atmospheric microplastic deposition in urban environments.

## 1. Introduction

Microplastics (plastic particles <5 mm in size) were first identified as contaminants in the environment during the 1970s [1]. In general, they are classified as either primary or secondary, i.e., manufactured to a microscopic size, or the degradation product of larger plastic particles via physical abrasion, biodegradation, and UV radiation, respectively [2,3,4]. As a result of the exponential growth in plastic waste, coupled with its persistence, microplastics have become ubiquitous within every environmental compartment globally [2,4,5]. However, most studies have primarily focused on aquatic (marine) environments, despite the growing evidence of the atmosphere as a transport pathway for microplastics (in particular, microfibres from textiles) in urban and remote environments [3,6,7,8,9,10]. In urban areas, the few studies that exist have observed microplastic atmospheric deposition to range from 10 microplastics (mp) m^−2^ day^−1^ (Gdynia, Poland; [11]) to 771 mp m^−2^ day^−1^ (Central London, UK; [9]). However, atmospheric sampling methods are generally labor-intensive, leading to spatially limited observations.

Biological monitors (biomonitors) are living organisms commonly used to determine the abundance or presence of an anthropogenic pollutant. Mosses have been used as biomonitors of atmospheric deposition since the late 1960s because they have a high capacity to trap and accumulate atmospheric particles [12,13,14,15]. Moss biomonitoring has been widely used to assess the atmospheric deposition of nitrogen [16,17,18], trace metals [15,19,20], persistent organic pollutants [21,22,23], and radionuclides [24,25,26]. Species such as *Pleurozium schreberi*, *Hylocomium splendens*, and *Hypnum cupressiforme* have been widely used for monitoring atmospheric deposition because they are broadly distributed (essentially found everywhere) and relatively easy to sample [27,28,29]. To date, one study has suggested that moss is also an effective biomonitor for atmospheric microplastic deposition [30]. Although moss species are widespread in natural areas, they are generally scarce in urban environments; as such, ‘active’ biomonitoring with moss bags (i.e., a mesh screen that encases a moss sample) is widely used in areas where naturally occurring species are unavailable [31,32].

The objective of this study was to assess the efficacy of moss bags as active biomonitors of atmospheric microplastic deposition. Moss bags (containing *Pleurozium schreberi* (Brid.) Mitt. (red-stemmed feathermoss) obtained from a rural background site) were deployed along a gradient of urban intensity in southern Ontario, Canada. It was predicted that moss bags exposed in the most densely populated and trafficked areas along the urban gradient would accumulate (with reference to unexposed moss bags) a higher concentration of microplastics (number of mp per g^−1^ dry weight (dw) moss) compared with low-density suburban areas.

## 2. Materials and Methods

### 2.1. Study Area and Study Sites

The study was carried out in southern Ontario, across an urban gradient from Peterborough City (PTB) through the Greater Toronto Area (GTA) along highway 401 to Downtown Toronto (TOR). The region experiences humid continental weather patterns consisting of harsh winters and strong seasonal variation. Long-term (1980–2010) annual precipitation is approximately 900 mm, and mean annual temperature is ~10 °C with winter lows below –30 °C and summer highs above 30 °C [33].

**Figure 1:**
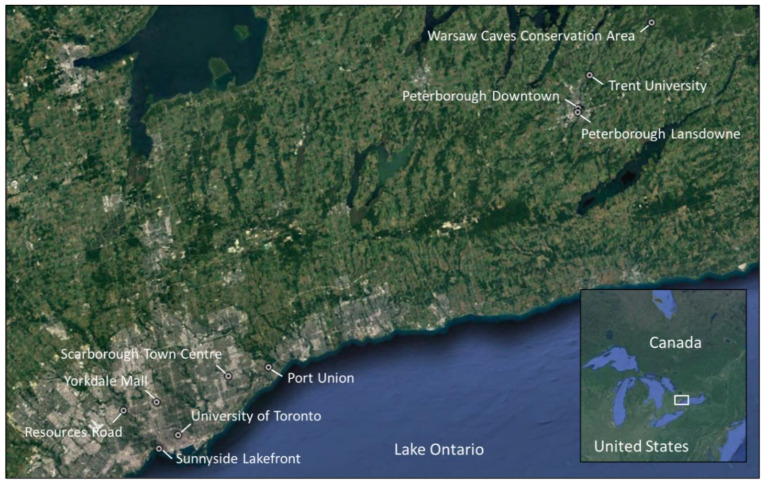
Location of the ten study sites; nine sites where moss bags were deployed from 9 October to 23 November 2020 (45 days) and the rural background site at Warsaw Caves Conservation Area where *Pleurozium schreberi* (Brid.) Mitt. (red-stemmed feather moss) was obtained for the moss bags (see Table 1). The inset shows the location of the study area in north America.

There were ten study sites; nine sites where moss bags were deployed, which were chosen to reflect a gradient in urban population and road traffic density, and one rural background site where the study moss was obtained (Figure 1 and Table 1). The GTA (inclusive of Toronto) has a population of 6 million, while Peterborough City, 125 km to the northeast, has a population of only ~81,000 [34]. Six sites were located within the GTA, which included four sites along highway 401 (the busiest highway in north America, with ~450,000 vehicles day^−1^) and two within the City of Toronto. Three sites were located within the City of Peterborough, two in high-traffic locations relative to city size (10,000–25,000 day^−1^; [35]), and one at Trent University, adjacent to a parking lot (100 spaces) close to student residences (Table 1). The rural background site was located at Warsaw Caves Conservation Area (CON), in Douro-Dummer township (population 7800), where moss was obtained for the production of all moss bags including unexposed (control) bags. The nine sites with moss bag deployments were further classified into three groups to reflect a gradient from low to high urban intensity based on population density and average daily traffic volumes (Table 1): PTB with traffic volumes up to 25,000 vehicles day^−1^ (*n* = 3; site ID 1, 2, and 3); TOR with traffic up to 80,000 vehicles day^−1^ (*n* = 2; site ID 8 and 9); and GTA with traffic up to 450,000 vehicles day^−1^ (*n* = 4; site ID 4, 5, 6, and 7).

### 2.2. Moss Bag Construction and Deployment

The moss species used in this study, *Pleurozium schreberi* (Brid.) Mitt., was collected from a rural background site, Warsaw Caves Conservation Area, on 18 September 2020 (Figure 1). This species was selected as it is widely used as a biomonitor of trace-element deposition [19,36]. This rural location was selected owing to its remoteness from human activity and distance (>100 m) from the nearest road. Using clean hands, moss (~5 g) was collected into brown paper bags (*n* = 5). The samples were collected from several beds of moss within a 50 m by 50 m area. Only the green parts of the moss were collected, as they represent the newest growth (two–three years). The samples were lightly cleaned of debris in the field and oven dried at 40 °C for 48 h upon return to the laboratory. Once dried, the moss was again cleaned of debris and weighed into 1 g sub-samples.

Moss bags (*n* = 23) were each made from a piece of 8 cm by 10 cm aluminium mesh screen with a 1 mm pore size folded onto itself to form a rectangular pocket (8 cm × 5 cm). Approximately 1 g of dried moss was added to the mesh pocket and sewn together on the three remaining sides using galvanized steel wire (28-gauge); each moss bag was individually wrapped in aluminium foil prior to deployment. The ratio between moss weight and bag surface area has been shown to influence uptake, with a recommended ratio < 15 mg cm^−2^ [37]; in this study, the ratio was 12.5 mg cm^−2^. In total, 23 moss bags were created; 18 were deployed as duplicates across the nine urban gradient sites (i.e., two moss bags per site) on 9 October 2020 for a six-week period, resulting in an average exposure of 1068 h (45 days). The duplicate bags were attached to zinc-plated L brackets and fastened to utility poles or light posts approximately three meters above the ground. Deployment sites were selected to ensure unobstructed airflow in all directions. The remaining five unexposed (control) moss bags were individually wrapped in aluminium foil and stored in paper envelopes for the duration of the exposure period. On November 23, moss bags were collected; wrapped in aluminium foil; returned to the laboratory, where all bags (including the unexposed controls) were dried at 40 °C for 48 h; and re-weighed to determine the mean change in moss biomass during the deployment period.

### 2.3. Digestion and Microplastic Extraction

Individual moss samples were digested using a wet peroxide (H_2_O_2_) oxidation method [38,39]. The content of each moss bag (~1 g), including the unexposed controls, was emptied into separate 500 mL glass beakers and digested using 40 mL of 0.05 M Fe (II) and 40 mL of 30% H_2_O_2_. Each digestate solution was left at room temperature for five minutes then added to a hot plate and heated to approximately 50 °C to increase the reaction [39]. Additional H_2_O_2_ aliquots were added in 20 mL increments when the reaction slowed down, and at least 100 mL of H_2_O_2_ was used for each sample. The mesh screen and aluminium foil from each moss bag were individually triple-rinsed with filtered B-pure water to capture microplastics potentially retained on their surfaces. Digested samples and their associated rinse water were then vacuum-filtered onto glass-fibre filter papers (Fisherbrand™ G6 (09-804-42A) 1.6 µm; three filters per sample; Pittsburgh, AR, USA), which were subsequently transferred to covered Petri dishes for storage until microplastic identification.

### 2.4. Microplastic Identification

Filter papers were visually analysed under a stereomicroscope with a digital camera attachment (Leica EZ4W with EZ4W0170 camera). In general, visual analysis is limited to particles >50 µm [40]. Microplastic particles were grouped into three categories: fibres, films, and fragments. The identification of microplastics followed well-established criteria [5,41,42]. Identification criteria for microplastic fibres included: (i) no cellular or organic structures visible; (ii) particles equally thick throughout the entire length; and (iii) particles exhibiting clear and homogenous colour throughout. Fragment and film identification criteria included: (i) no cellular or organic structures visible; (ii) irregular shape; and (iii) unnatural colouration. Particles resembling microbeads were quantified as fragments, as they represented a very small fraction of the observed microplastics. Particles that passed visual inspections were prodded using tweezers; most plastic pieces are flexible and will bounce and spring when prodded [41]. Particles that did not break were photographed for subsequent measurement. Microplastics were further verified using a hot needle test [43,44]; if a particle melted or curled under the presence of a hot needle, it was counted as a microplastic. If it did not react, it was not counted as a microplastic (and the photograph was deleted), as the particle was likely another anthropogenic material such as a cotton fibre. Tire particles (counted as fragments) are more difficult to identify, as they do not react to the hot needle test, and so they were classified using specific criteria: (i) darkly coloured (black); (ii) elongated or cylindrical in shape; (iii) rough surface texture; and (iv) rubbery flexibility when manipulated [45,46,47]. Potential tire fragments were required to meet all four identification criteria to be classified as a microplastic.

### 2.5. Quality Control

Strict quality-control procedures were followed to ensure that contamination was minimized during sampling and laboratory analysis (see [48]). Moss bags were wrapped in aluminium foil outside of their deployment period. All B-pure water was vacuum-filtered prior to cleaning glassware and use in the extraction process. All laboratory glassware used during digesting and filtering was covered with aluminium foil to prevent airborne contamination, and all glassware was rinsed in triplicate with filtered B-pure water. Surfaces (bench, fume hood, sink, etc.) were wiped down with paper towels and B-pure between the digestion of each sample. Procedural open-air blanks (average exposure time of 5 h) were used to determine the amount of potential contamination during sample digestion, filtration, and identification stages. Digestion blanks were vacuum-filtered using 50 mL filtered B-pure in place of sample media and analysed for microplastic contamination. Peroxide and Fe (II) solution blanks (1 L, respectively) were also filtered and analysed for microplastics. Finally, cotton clothing was worn during the collection of moss, production of moss bags, and laboratory extraction of microplastics.

### 2.6. Data Analysis

Microplastic particle counts (mp) for fibres, fragments, and films were summed to estimate the total number per bag; count concentration (mp g^−1^) was estimated by dividing the microplastic count per bag by its respective moss dry weight. The level of detection (LOD) was estimated as the mean microplastic count for the five unexposed (control) moss bags plus three times their standard deviation. Moss bags with microplastic counts below the LOD were identified but not removed from the analysis; our goal was to evaluate the efficacy of moss bags as active biomonitors rather than assess microplastic deposition. Variation between duplicate bags at each deployment site was estimated as relative percent difference (RPD). The counts observed at each deployment site were averaged across the duplicate bags; this mean was used to calculate the number of microplastic particles accumulated during the exposure period, i.e., the mean count for exposed moss bags (*n* = 2) minus the mean of the unexposed control bags (*n* = 5). These data were used to estimate daily microplastic deposition (mp m^−2^ day^−1^) based on the exposure period (45 days) and surface area of the moss bag (5 cm × 8 cm = 0.004 m^2^).

The nine deployment sites were combined into three groupings (PTB, GTA, and TOR; see Table 1) to assess the accumulation of microplastics in relation to the gradient of urban intensity, which was based on population and traffic density. Microplastic particles were measured in ImageJ open-source software to determine length and width. Median rather than mean length is presented, as the data were not normally distributed (e.g., see [3]); the median is a better measure of the central tendency for positively skewed data. Length and width were further used to estimate microplastic particle volume per moss bag (mm^3^ g^−1^); fibre volume was estimated as a cylinder, film volume as a rectangular prism, and fragment volume as an ellipsoid. Microplastics identified in procedural blanks (open air and digestion) were averaged to estimate potential contamination. However, microplastic counts were not blank-corrected, as accumulation was estimated as the difference between exposed and unexposed (control) moss bags, which underwent the same analytical procedures. Statistical differences in microplastic counts (per moss bag) across the urban intensity gradient groups were assessed using a Kruskal–Wallis test followed by a Mann–Whitney U pairwise test (PAST 4.11; [49]).

## 3. Results

During the deployment period (45 days; 9 October–23 November 2020), the mean temperature across the study area was 7.3 °C (range: −9.9 °C to 24.8 °C), and the mean total precipitation was 44.0 mm (see Supplementary Materials Table S1). There was a slight reduction (~6%) in the mass of moss per bag during exposure, i.e., the average mass per bag was 0.95 g following collection. Potential contamination estimated from digestion and open-air blanks was approximately 0.65 microplastics per moss bag (see Supplementary Materials Table S2). However, samples were not blank-corrected, as microplastic accumulation by moss bags was estimated by subtracting unexposed (control) counts from exposed counts, which accounted for potential contamination.

Microplastics were observed in all moss bags (*n* = 23), including exposed and unexposed (control) bags (see Supplementary Materials Figure S1 and Table S3). In total, 200 microplastics were observed across the ten sites: 186 particles in the exposed bags (*n* = 18), and 14 particles in unexposed bags (*n* = 5). Mean counts at the study sites ranged from 5 (Trent University) to 17 (Resources Road), with 2.8 microplastics in the unexposed (control) bags (see Table 2). The LOD estimated from the five unexposed bags was 6.3 mp; only three of the eighteen exposed moss bags (nine sites with duplicate exposures) were below this level, two at Peterborough Lansdowne and one at Trent University (see Supplementary Materials Table S3). Nonetheless, mean counts at the deployment sites were greater than the mean unexposed (Warsaw Caves) control (Table 2). The mean variation in counts between duplicate moss bags across the deployment sites was 29% and ranged from 9% (Yorkdale Mall) to 80% (Trent University). The mean microplastic accumulation across all exposed bags (*n* = 18) during the study period was 7.9 mp g^−1^ (dw moss), ranging from 2.5 mp g^−1^ (Trent University) to 15.0 mp g^−1^ (Resources Road; see Table 2).

**Figure 2:**
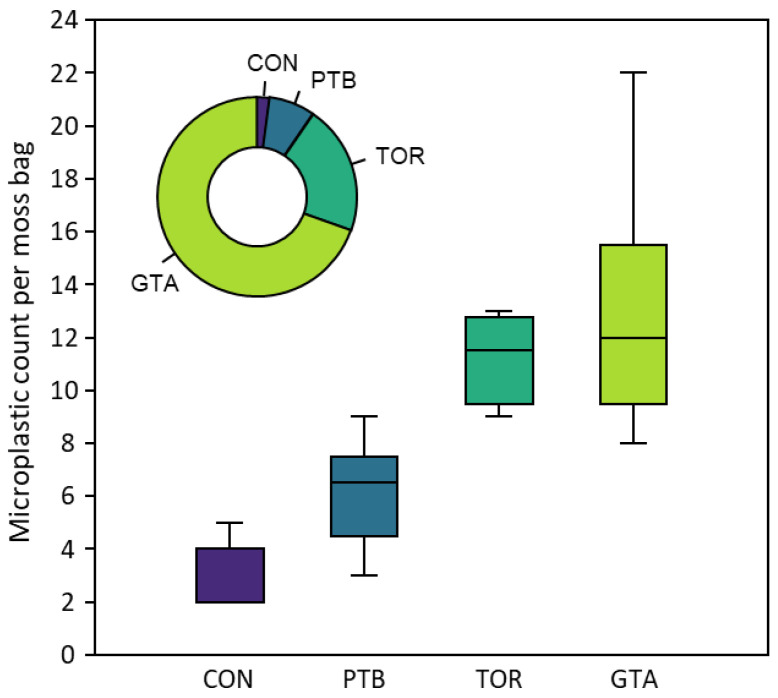
Boxplot showing microplastic count per moss bag within the three urban intensity groups (PTB, TOR, and GTA) and the unexposed control (CON) during the deployment period (45 days, 9 October–23 November 2020). The inset doughnut chart shows the mean microplastic volume per moss bag across the four groups (see Table 3).

Across the urban intensity gradient, the mean microplastic counts ranged from 6.2 (PTB) to 13 (GTA) per group (see Table 3 and Figure 2). Moreover, the mean microplastic volume per moss bag showed a greater separation between groups, ranging from 0.007 mm^3^ g^−1^ (PTB) to 0.019 mm^3^ g^−1^ (TOR) and 0.064 mm^3^ g^−1^ (GTA), compared with 0.002 mm^3^ g^−1^ in the unexposed (control) moss bags (Figure 2). The mean microplastic accumulation per group during exposure ranged from 3.7 mp g^−1^ (PTB) to 8.8 mp g^−1^ (TOR) and 10.7 mp g^−1^ (GTA). There was a significant difference in accumulation across the groups (Kruskal–Wallis, *p* < 0.01); the microplastic concentration was significantly greater at GTA and TOR compared with PTB (Mann–Whitney U, *p* < 0.05), but GTA and TOR were not significantly different. Further, microplastic counts for all groups were significantly different (higher) compared with the unexposed (control) bags (see Figure 2). The atmospheric deposition of microplastics within each group was estimated to be 21 mp m^−2^ day^−1^ in PTB, 50 mp m^−2^ day^−1^ in TOR, and 60 mp m^−2^ day^−1^ in GTA (Table 3).

**Figure 3:**
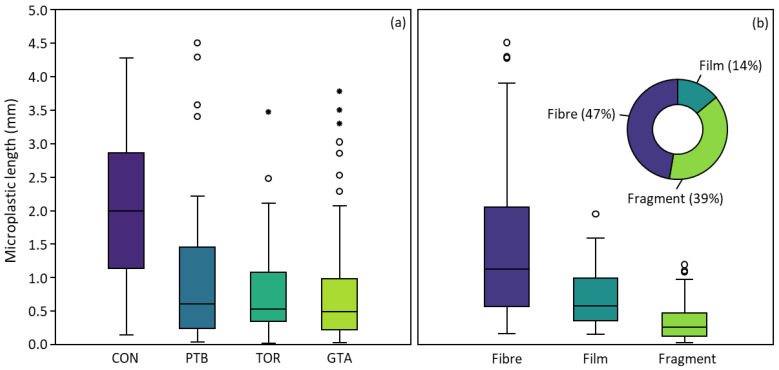
Boxplots showing the distribution of microplastic length in moss bags (**a**) deployed across the three urban intensity groups (PTB, TOR, and GTA) and in the unexposed (control) moss bags (CON), and (**b**) by particle type (fibre, fragment, or film) across all exposed and unexposed moss bags during the deployment period (45 days; 9 October–23 November 2020). The inset (**b**) doughnut chart shows the percentage of total microplastics composed of fibres (47%), fragments (39%), and films (14%). Note: four microfibres >5 mm were removed from the dataset.

The median microplastic length was 0.56 mm (range 0.03–4.51 mm) across all moss bags; four microplastics (fibres) were larger than 5 mm and were removed from the dataset (see Supplementary Materials Table S4). Median microplastic length varied little between urban intensity groups, ranging from 0.49 mm (GTA) to 0.53 mm (TOR) and 0.61 mm (PTB). In contrast, the median microplastic length was significantly higher (2.00 mm; Mann–Whitney U, *p* < 0.01) in the unexposed (control) moss bags (Figure 3). Similarly, there was a significant difference in length between microplastic types (Kruskal–Wallis, *p* < 0.001) in the order of fibres (median 1.13 mm) > films (0.57 mm) > fragments (0.26 mm) across all moss bags (Figure 3; Mann–Whitney U, *p* < 0.001).

Microfibres were the dominant shape (47%), followed by fragments (39%) and films (14%), across all sites (Figure 3). In contrast, only ~5% of all microplastics in the exposed moss bags were identified as tire fragments and 3% as beads (both included in the fragment category), suggesting that these particles settle more rapidly from the atmosphere than other particle types. The percentage of microfibres differed greatly across the urban intensity groups (Table 3), ranging from 33% (TOR) to 62% (PTB), with fibres dominant in low-density suburban areas. Further, fibres made up 93% of microplastics in the unexposed (control) moss bags.

## 4. Discussion

Microplastics were observed in all exposed moss bags across the deployment sites (*n* = 18) and in moss from the rural background site (Table 2). Further, there was a significant difference (increase) in microplastic counts in moss bags with increasing urban intensity across the deployment sites, i.e., the accumulation of microplastics at the TOR and GTA sites (8.8 and 10.7 mp g^−1^) was significantly higher than at PTB (3.7 mp g^−1^), which was the lowest-intensity urban area (Table 3). The ability of moss to entrap and retain microscopic particles implies that moss bags may be a reliable active biomonitor of atmospheric microplastic deposition in urban areas. Nonetheless, few studies have evaluated biological monitoring as a technique for assessing the atmospheric deposition of microplastics.

One study assessed the deposition of plastic microfibres (mf) in natural moss beds of *Hylocomium splendens* across three rural background sites in Ireland [30]; the mean concentration across the three sites was 3.1–6.4 mf g^−1^, and the estimated microfibre deposition was 6.8–14.1 mf m^−2^ day^−1^. Similar levels of microplastics were observed at the rural background and suburban locations in the current study; the microfibre concentration at Warsaw Caves was 2.7 mf g^−1^, and the microfibre deposition was 13 mf m^−2^ day^−1^ across PTB (Table 3). Several studies have evaluated atmospheric microplastics in lichens [8,50]; one study used epiphytic lichens (*Flavoparmelia caperata*) along a gradient of varying proximity to a landfill in northern Italy [8]. The microplastic concentration at the most remote site (1.5 km from the landfill) ranged from 3 to 9 mp g^−1^, which was similar to Warsaw Caves (2.9 mp g^−1^). Another study used transplants of the fruticose lichen *Evernia prunastri* (exposed in triplicate for three months) in the urban area of Milan and at a background control site in northern Italy [50]. Microplastic deposition at the background control site (50 km north of Milan) ranged from 21 to 43 mp m^−2^ day^−1^, which was similar to PTB (21 mp m^−2^ day^−1^), 125 km northeast of Toronto. Further, microplastic deposition across Milan ranged from 43 to 119 mp m^−2^ day^−1^, which was consistent with the 50–60 mp m^−2^ day^−1^ range observed across the TOR and GTA urban intensity groups in the current study (Table 3).

Fibres appear to dominate microplastic deposition (for particles > 50 µm) at sites remote from urban centres (e.g., [6]), suggesting that fibres may be subject to prolonged atmospheric suspension (or fragments are subject to more rapid deposition). Overall, 47% of all microplastics in the moss bags were identified as fibres (Figure 3). Fibres were the dominant particle type (93%) observed at Warsaw Caves (CON), and their dominance increased with decreasing urban intensity from 33% (TOR) to 62% (PTB), suggesting that fibres are more prone to long-range transport given their greater surface-area-to-volume ratio, which increases drag force and reduces settling velocity [6]. Similarly, in northern Italy, the percentage of microfibres observed in the lichen *Flavoparmelia caperata* increased with distance from a landfill, i.e., from 41% facing the landfill to 73% at a distance of 1.5 km away [8].

There are a growing number of studies suggesting that moss and lichen are effective biomonitors of atmospheric microplastic deposition, suitable for use as active biomonitors across urban environments. Nonetheless, there are a number of unknowns that require further study, such as the mechanism of microplastic adsorption (e.g., entrapment, electrostatic attraction, etc.); microplastic retention efficiency and capacity; and the influence of moss bag design (bag size, mesh size, and moss species). Ideally, future studies should also evaluate the performance of passive and active biomonitoring of microplastic deposition against traditional atmospheric monitoring techniques.

## 5. Conclusions

This is the first study to evaluate the use of moss bags for active biomonitoring of atmospheric microplastic deposition. Atmospheric microplastics were observed across all moss bags deployed in southern Ontario. The magnitude of microplastics accumulated during the 45 days of deployment suggests that moss bags are effective biomonitors of atmospheric microplastic deposition in urban areas. Further, deployment along the urban gradient suggested that more densely populated and trafficked areas have greater rates of atmospheric microplastic deposition. Finally, the variation in microplastic particle type across the urban gradient suggests that fibres are likely to be dominant at sites farther from sources.

## Figures and Tables

**Table 1:** Site ID; group (urban intensity); name; annual average daily traffic (AADT, vehicles day^−1^); and coordinates (latitude and longitude, decimal degrees) for the nine deployment sites and the rural background site in southern Ontario, Canada (*n* = 10).

ID	Group	Site Name	AADT	Latitude	Longitude
1	PTB	Peterborough Lansdowne	24,900	44.288033	–78.320410
2	PTB	Peterborough Downtown	10,400	44.295368	–78.319133
3	PTB	Trent University	<200	44.359686	–78.287522
4	GTA	Port Union	233,900	43.796374	–79.154208
5	GTA	Scarborough Town Centre	315,900	43.779017	–79.262005
6	GTA	Yorkdale Mall	397,000	43.727960	–79.454669
7	GTA	Resources Road	442,900	43.711166	–79.543352
8	TOR	Sunnyside Lakefront	77,000 ^§^	43.637672	–79.447855
9	TOR	University of Toronto St. George	17,800 ^$^	43.664392	–79.396623
10	CON	Warsaw Caves Conservation Area	130	44.460905	–78.117738

^§^ Adjacent to the Gardiner Expressway, which has an AADT of ~169,000 vehicles day^−1^; ^$^ the surrounding city block has an AADT range of ~35,000–68,000 vehicles day^−1^.

**Table 2:** Mean microplastic count, relative percent difference (RPD) between duplicate moss bags, and percent fibres for each study site including the rural background site, and accumulated microplastic concentration (g^−1^ dry weight moss) in moss bags during the deployment period (9 October–23 November 2020). Accumulation = exposed moss bags (mean of duplicate)—unexposed moss bags (mean of five controls), i.e., microplastics captured during the exposure period at each site.

ID	Study Site	Count	RPD	%Fibre	mp g^−1^
1	Peterborough Lansdowne	5.5 ^$^	18	55	2.8
2	Peterborough Downtown	8.0	25	69	5.7
3	Trent University	5.0 ^$^	80	60	2.5
4	Port Union	15.0	13	50	12.9
5	Scarborough Town Centre	8.5	12	71	5.8
6	Yorkdale Mall	11.5	9	30	9.2
7	Resources Road	17.0	59	47	15.0
8	Sunnyside Lakefront	10.5	29	43	8.1
9	University of Toronto	12.0	17	25	9.5
10	Warsaw Caves ^§^	2.8	47	93	

^§^ Variation between five control bags shown as relative standard deviation; ^$^ microplastic counts < level of detection (LOD = 6.3 mp based on unexposed (control) moss bags from Warsaw Caves).

**Table 3:** Mean microplastic count, percent fibre, mean microplastic volume in the unexposed control (CON) bags and three urban intensity groups (PTB, TOR, and GTA), and accumulated concentration (mp g^−1^) in moss bags (dry weight) during the deployment period (45 days; 9 October–23 November 2020). Estimated daily deposition of microplastics (mp) and plastic microfibres (mf) are also shown.

Group	Microplastic Count	Fibre %	Volume mm^3^ g^−1^	Concentration mp g^−1^ (45 Days)	Deposition mp m^−2^ Day^−1^	Deposition mf m^−2^ Day^−1^
CON	2.8	93	0.002			
PTB	6.2	62	0.007	3.7	21	13
TOR	11.3	33	0.019	8.8	50	17
GTA	13.0	48	0.064	10.7	60	29

## Data Availability

The data used in this study are provided in the Supplementary Materials.

## Supplementary Materials

The following supporting information can be downloaded at: https://www.mdpi.com/article/10.3390/biology12020149/s1, Figure S1: Examples of microplastics identified in moss bags, Table S1: Climate data from the nearest meteorological monitoring stations to deployed moss bags, Table S2: Count and length (mm) of microplastic particles found in blanks, Table S3: Count of fibres, fragments, and films in each moss bag, Table S4: Particle type, length (L), and width (W) of microplastic particles (mm) found in moss bags across the urban intensity groups.
