# Supplementary material for: Moss Bags as Biomonitors of Atmospheric Microplastic Deposition in Urban Environments
Source: Biology (Basel). 2023 Jan 18;12(2):149. doi: 10.3390/biology12020149 (PMC9953122; doi:10.3390/biology12020149)
Supplement: Supplementary file 1 [file biology-12-00149-s001.zip › biology-2168073-supplementary.pdf]

Supplementary Materials

Moss bags as biomonitors of atmospheric microplastic deposition in urban environments

Carter Bertrim and Julian Aherne *

School of Environment, Trent University, Peterborough, ON K9L 0G2, Canada

* Correspondence: jaherne@trentu.ca

Supplementary Materials: Figures and Tables

Figure S1. Examples of microplastics identified in moss bags (a) dark coloured fibre from Lansdowne Peterborough (PTB), (b) dark coloured fibre from Port Union (GTA), (c) red coloured fragment from Resources Road (GTA), (d) dark coloured film from Sunnyside Lakefront (TOR), bright blue film from University of Toronto St. George (TOR), (f) white microplastic bead from undeployed moss bag (CON). Beads such as this (one of three in total) were quantified as microplastic fragments for the purpose of this study. Material stuck to microplastics is residual organic material post-digestion.

Table S1. Climate data from the nearest meteorological monitoring station to deployed moss bag groups during the study period October 9 to November 23, 2020 (45 days). Temperature in the PTB site grouping ranged from –9.9°C to 24.8°C with 31.0 mm of precipitation over 19 days, GTA ranged from –7.5°C to 24.5°C with 64.1 mm of precipitation over 29 days, and TOR ranged from –4.8°C to 22.8°C with 37.0 mm of precipitation over 22 days.

Table S2. Count and length (mm) of microplastic particles found in procedural open-air blanks, digestion blanks, H_2O_2, B-Pure, and Fe (II) solution blanks. This potential contamination from H_2O_2, B-Pure, and Fe (II) solutions was eliminated via filtration. Open-air blanks were used through all steps where samples were vulnerable to exposure (average exposure time 5 hours).

Table S3. Count of fibres, fragments (including tire fragments), and films in each moss bag (see Table 1 for details on Site ID). The dry weight (g) of moss added to each bag pre-deployment and a subset for post-deployment is also given.

Table S4. Particle type, length (L), and width (W) of microplastic particles (mm) found in moss bags across the Peterborough (PTB, n = 6), Greater Toronto Area (GTA, n = 8), Toronto (TOR, n = 4), and Warsaw Caves Conservation Area (CON, n = 5) urban intensity groups.

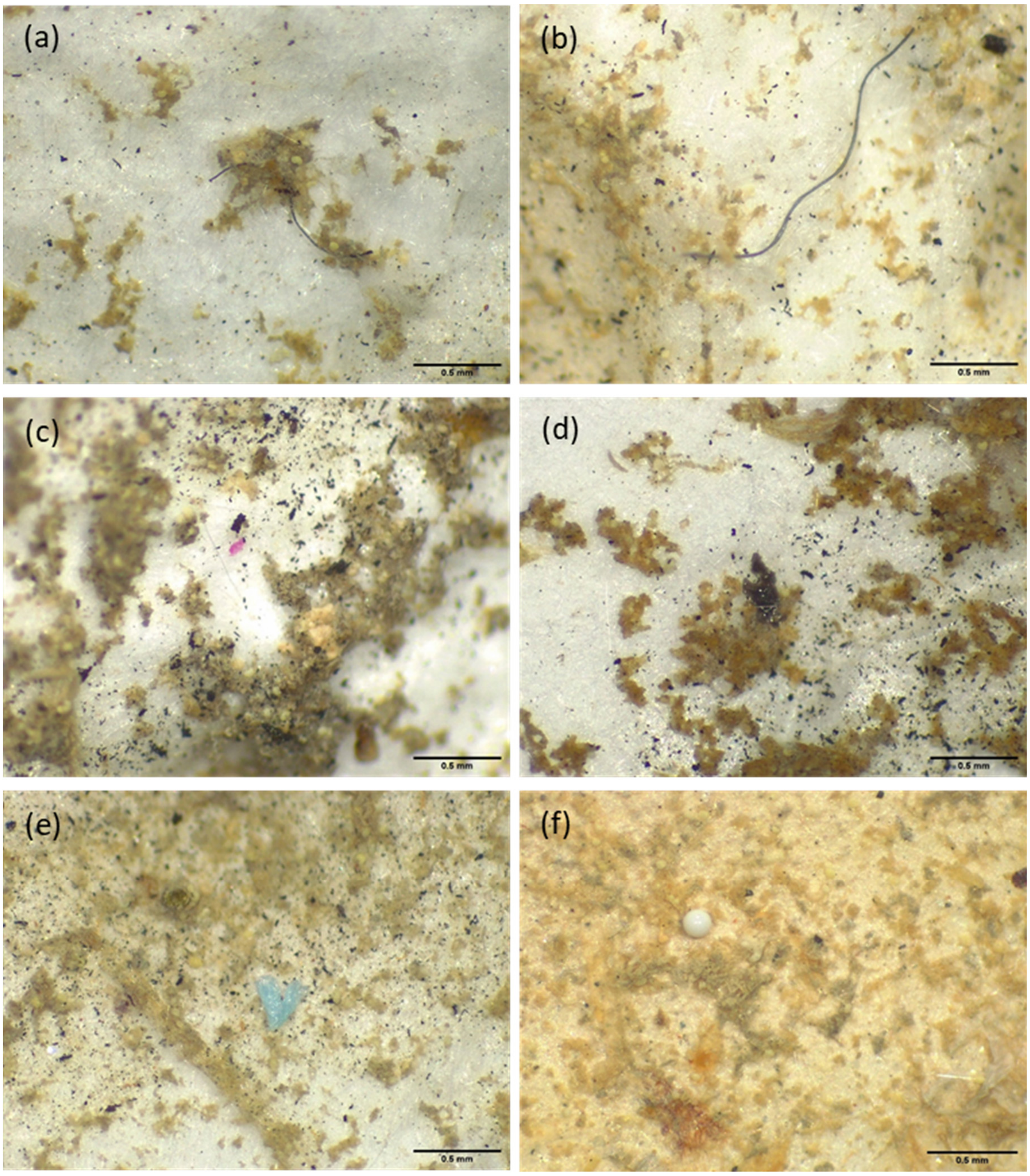

Figure S1. Examples of microplastics identified in moss bags (a) dark coloured fibre from Lansdowne Peterborough (PTB), (b) dark coloured fibre from Port Union (GTA), (c) red coloured fragment from Resources Road (GTA), (d) dark coloured film from Sunnyside Lakefront (TOR), bright blue film from University of Toronto St. George (TOR), (f) white microplastic bead from undeployed moss bag (CON). Beads such as this (one of three in total) were quantified as microplastic fragments for the purpose of this study. Material stuck to microplastics is residual organic material post-digestion.

Table S1. Climate data from the nearest meteorological monitoring station to deployed moss bag groups during the study period October 9 to November 23, 2020 (45 days). Temperature in the PTB site grouping ranged from –9.9°C to 24.8°C with 31.0 mm of precipitation over 19 days, GTA ranged from –7.5°C to 24.5°C with 64.1 mm of precipitation over 29 days, and TOR ranged from –4.8°C to 22.8°C with 37.0 mm of precipitation over 22 days.

Station Name	PTBO	GTA	TOR	Average
Latitude (°)	44.23000	43.78000	43.67000	
Longitude (°)	–78.36000	–79.47000	–79.40000	
Mean Temperature (°C)	5.79	7.29	8.84	7.31
Min Temperature (°C)	–9.9	–7.5	–4.8	–7.4
Max Temperature (°C)	24.8	24.5	22.8	24.0
Total Precipitation (mm)	30.97	64.09	36.96	44.01
Days of Precipitation (count)	19	29	22	

Table S2. Count and length (mm) of microplastic particles found in procedural open-air blanks, digestion blanks, H_2O_2, B-Pure, and Fe (II) solution blanks. This potential contamination from H_2O_2, B-Pure, and Fe (II) solutions was eliminated via filtration. Open-air blanks were used through all steps where samples were vulnerable to exposure (average exposure time 5 hours).

Blanks	Number	Particle	Length (mm)
Air	1	1	0.9
	1	2	2.487
	2	0	–
	3	1	0.509
	4	0	–
Digestion	1	1	0.434
	1	2	0.423
	2	0	–
	3	0	–
	4	0	–
H_2O_2	1	0	–
	2	1	0.082
	2	2	0.311
B-Pure	1	1	1.186
	2	0	–
Fe (II)	1	1	1.026
	1	2	0.489
	1	3	3.292
	2	0	–

Table S3. Count of fibres, fragments (including tire fragments), and films in each moss bag (see Table 1 for details on Site ID). The dry weight (g) of moss added to each bag pre-deployment and a subset for post-deployment is also given.

Site	Bag	Pre weight (g)	Post-weight (g)	Fibres	Fragments	Tire	Films	Total
1	1	1.0617	0.9165	4	0	1	1	6
1	2	0.9845	0.8674	2	2	1	0	5
2	3	0.9815		6	0	0	1	7
2	4	0.9954		5	3	0	1	9
3	5	0.9891		2	1	0	0	3
3	6	0.9945		4	3	0	0	7
4	7	1.0120	0.876	9	7	0	0	16
4	8	1.0105		6	7	0	1	14
5	9	1.0054	0.9129	7	1	0	0	8
5	10	1.0600		5	4	0	0	9
6	11	1.0220		1	8	0	3	12
6	12	1.0074		6	4	1	0	11
7	13	1.0012	1.2255	11	11	0	0	22
7	14	1.0194		5	3	0	4	12
8	15	1.0271	9.7267	7	2	0	3	12
8	16	1.0017		2	2	1	4	9
9	17	1.0303	0.9119	5	2	1	5	13
9	18	1.0211		1	4	3	3	11
10	19	1.0536		3	0	0	0	3
10	20	1.0035		2	0	0	0	2
10	21	1.0029		4	1	0	0	5
10	22	1.0435		2	0	0	0	2
10	23	1.0562		2	0	0	0	2

Table S4. Particle type, length (L), and width (W) of microplastic particles (mm) found in moss bags across the Peterborough (PTB, n = 6), Greater Toronto Area (GTA, n = 8), Toronto (TOR, n = 4), and Warsaw Caves Conservation Area (CON, n = 5) urban intensity groups.

PTB (mm)			GTA (mm)			TOR (mm)			CON (mm)	
Type	L	W	Type	L	W	Type	L	W	Type	L
fr	0.954		fr	0.868		fr	0.468		fr	2.045
fr	1.19		fr	0.679		fr	0.79		fr	1.999
fr	0.456		fr	0.471		fr	2.001		fr	3.898
fr	0.366		fr	5.878		fr	2.482		fr	0.67
fr	0.854		fr	2.072		fr	0.379		fr	2.42
fr	0.632		fr	1.548		fr	1.781		fr	4.277
fr	1.508		fr	1.818		fr	1.122		fr	1.051
fr	0.251		fr	0.222		fr	1.141		fr	5.402
fr	1.032		fr	3.504		fr	3.476		fr	1.487
fr	4.506		fr	2.856		fr	0.313		fr	2.364
fr	1.621		fr	0.526		fr	0.575		fr	3.3
fr	2.219		fr	0.785		fr	1.607		fr	1.232
fr	0.479		fr	1.079		fr	2.116		fr	1.593
fr	0.522		fr	2.289		fm	0.189	0.137	fg	0.144
fr	4.295		fr	0.289		fm	1.207	0.706		
fr	1.797		fr	1.013		fm	0.584	0.395		
fr	2.047		fr	1.577		fm	0.09	0.046		
fr	0.965		fr	0.546		fm	0.631	0.033		
fr	7.593		fr	12.307		fm	0.12	0.065		
fr	3.409		fr	0.256		fm	0.087	0.066		
fr	1.249		fr	3.784		fm	0.097	0.085		
fr	3.582		fr	1.221		fm	0.091	0.048		
fr	1.41		fr	1.134		fm	0.033	0.03		
fm	0.272	0.109	fr	0.82		fm	0.731	0.028		
fm	0.122	0.019	fr	0.591		fm	0.062	0.052		
fm	0.055	0.05	fr	0.877		fm	0.115	0.071		
fg	0.443	0.443	fr	0.564		fm	0.073	0.037		
fg	0.094	0.048	fr	1.031		fm	0.108	0.072		
fg	0.098	0.016	fr	0.49		fg	0.446	0.266		
fg	0.221	0.045	fr	0.287		fg	0.675	0.109		
fg	0.261	0.024	fr	3.302		fg	1.154	0.4		
fg	0.63	0.316	fr	0.665		fg	0.297	0.27		
fg	0.318	0.047	fr	0.158		fg	0.151	0.068		
fg	0.946	0.292	fr	0.245		fg	0.378	0.263		
fg	0.31	0.039	fr	0.37		fg	0.428	0.143		
fg	0.206	0.027	fr	0.422		fg	0.811	0.045		

fg	0.526	0.244	fr	1.997		fg	0.648	0.433
fg	0.456	0.319	fr	0.683		fg	0.425	0.245
			fr	1.176		fg	0.238	0.219
			fr	0.537		fg	0.349	0.033
			fr	3.026		fg	1.588	0.161
			fr	2.531		fg	0.923	0.108
			fr	0.683		fg	0.988	0.047
			fm	0.14	0.079			
			fm	0.254	0.12			
			fm	0.038	0.038			
			fm	0.124	0.045			
			fm	0.121	0.069			
			fm	0.229	0.037			
			fm	0.177	0.022			
			fm	0.334	0.031			
			fm	0.605	0.097			
			fg	0.419	0.365			
			fg	1.1	0.456			
			fg	0.411	0.27			
			fg	0.32	0.029			
			fg	0.113	0.03			
			fg	0.184	0.184			
			fg	0.252	0.016			
			fg	0.18	0.103			
			fg	1.191	0.005			
			fg	0.169	0.115			
			fg	0.31	0.306			
			fg	0.036	0.036			
			fg	0.155	0.027			
			fg	0.133	0.098			
			fg	0.342	0.258			
			fg	0.544	0.418			
			fg	0.549	0.025			
			fg	0.85	0.153			
			fg	0.306	0.038			
			fg	0.223	0.101			
			fg	0.573	0.221			
			fg	0.294	0.251			
			fg	0.459	0.184			
			fg	0.471	0.246			
			fg	1.948	0.304			

	fg	1.272	0.302		
	fg	0.796	0.428		
	fg	1.447	0.533		
	fg	0.392	0.029		
	fg	0.3	0.015		
	fg	0.025	0.025		
	fg	0.122	0.122		
	fg	0.308	0.058		
	fg	0.736	0.095		
	fg	0.295	0.041		
	fg	0.532	0.023		
	fg	1.08	0.146		
	fg	0.971	0.027		
	fg	0.624	0.089		
	fg	0.099	0.045		
	fg	0.51	0.196		
	fg	0.467	0.052		
	fg	0.417	0.095		
